# Supplementary material for: Interpretable machine learning for cardiovascular risk prediction: Insights from NHANES dietary and health data
Source: PLoS One. 2025 Nov 6;20(11):e0335915. doi: 10.1371/journal.pone.0335915 (PMC12591444; doi:10.1371/journal.pone.0335915)
Supplement: S1 File — Calibration curves before and after post-hoc calibration (S1 Fig); LIME explanations for the undersampled model (S2 Fig); and test-set discrimination and calibration metrics (S1 Table). (PDF) [file pone.0335915.s001.pdf]

Supporting Information

S1. What we mean by calibration

Calibration tells us whether the predicted probabilities behave like probabilities. If we group people by their predicted risk and compare it to what actually happened, well-calibrated models will lie on the 45° line: when the model says 20%, about 20% of people in that group should have the outcome.

We checked calibration on the untouched test set in two ways: (i) a reliability curve (mean predicted probability vs. observed rate by bin), and (ii) the Brier score (lower is better). We also show a post-hoc logistic calibration (Platt scaling) fit on a validation split from the training data; this mapping changes probabilities but does not change AUC.

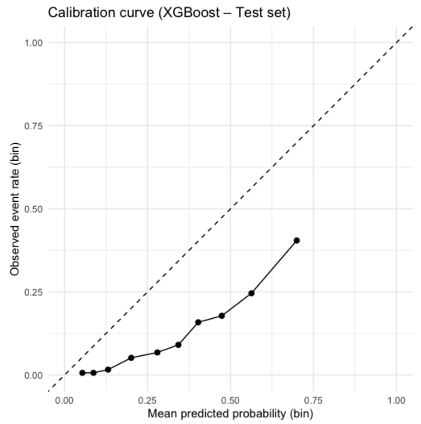

(a) Test-set calibration *before* post-hoc calibration. Dashed line = perfect calibration. Points below the line indicate over-prediction.

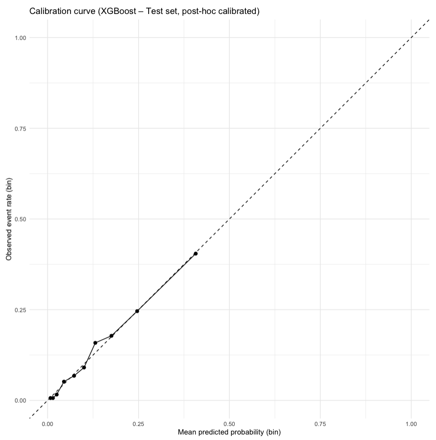

(b) Test-set calibration *after* post-hoc logistic calibration (Platt scaling). Points lie closer to the 45° line. Discrimination (AUC) is unchanged.

Figure 1: **Supplementary Fig. S1.** Calibration curves on the untouched test set, before (A) and after (B) post-hoc calibration.

Notes. These plots summarize probability quality. They complement, but do not replace, discrimination metrics such as AUROC.

S2. LIME examples for the undersampled model

LIME explains why the model made a prediction for one person at a time. Each small panel is one test case. Label is the class we explain (0 = non-CVD, 1 = CVD). The reported Probability is for that label (e.g., if Label = 0 and Probability = 0.66, that means $P(\text{non-CVD}) = 0.66$). Bars show which features pushed the prediction toward the label (blue) or away from it (red) for that person; longer bars mean a bigger push. We show borderline cases near the decision threshold, so probabilities cluster near 0.5. Explanation Fit means "how well the simple, local explanation matches the model for this case"; it is not an accuracy score.

S3. Test-set discrimination and calibration (numbers)

For completeness, we report AUROC and the Brier score on the untouched test set for both training strategies. Lower Brier indicates better probability quality.

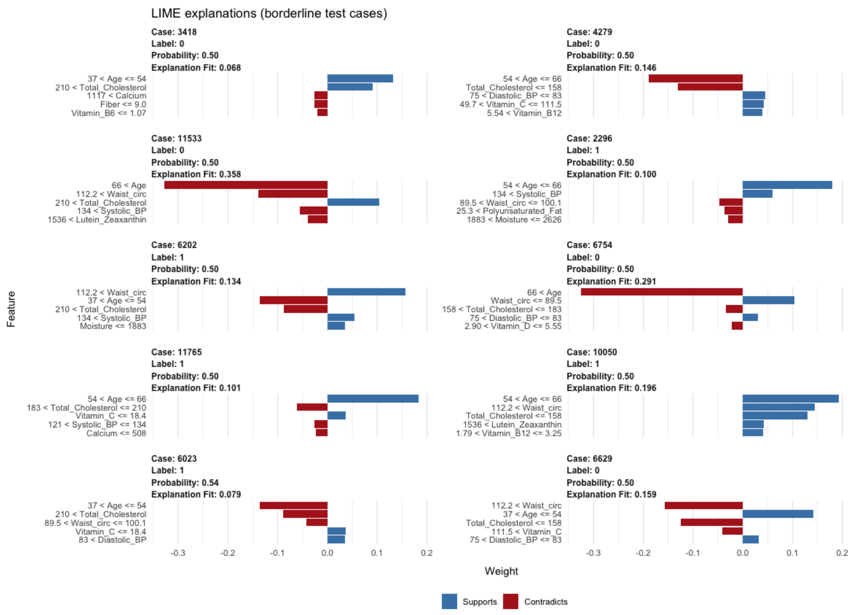

Figure 2: **Supplementary Fig. S2.** LIME explanations for the model trained with real-case undersampling. Feature directions mirror those in the main text (e.g., Age, Waist_circ, and blood pressure push risk up; lower values push risk down), supporting that LIME patterns are robust to the class-balancing method.

Table 1: **Test-set discrimination and calibration**

**Method**	**Train (n)**	**Test (n)**	**AUROC (Test)**	**Brier (Test)**
ROSE oversampling	9,287	3,095	0.802	0.141
Undersampling (real cases only)	2,278	3,095	0.792	0.207

Note. The post-hoc calibrated curve is shown in Fig. S1B for visualization. We do not report post-calibration coefficients here; AUC is unchanged by calibration.
