# Supplementary material for: Interpretable machine learning for cardiovascular risk prediction: Insights from NHANES dietary and health data
Source: PLoS One. 2025 Nov 6;20(11):e0335915. doi: 10.1371/journal.pone.0335915 (PMC12591444; doi:10.1371/journal.pone.0335915)
Supplement: Attachment — Submitted filename: PLOS ONE Review Report.pdf [file pone.0335915.s002.pdf]

Critical Concerns:

Fig 4: I understand this may just be a sample for demonstration purposes, but the low probabilities reported are concerning and suggests poor model performance or perhaps poor feature selection. Indeed, the model may be underfitting as features such as medications and other traditional comorbid CVD predictors were not included. Probabilities of 0.51 -0.57 are essentially random chance. This is a MAJOR shortcoming.

I am having a difficult time reconciling this with your ROC comparisons **in Fig 3.** If the models have AUC ~0.80, what are the individual prediction probabilities so low? Do you have other LIME cases that may be more representative? I suspect that this may be a class imbalance issue which your ROSE technique failed to satisfactorily resolve. There were only 1,518 CVD cases. You used ROSE to synthetically balance the classes to arrive at 4,592 CVD cases and 4,592 non-CVD cases in the training data. I wonder how the models will perform and how the LIME would look if you included only the actual 1,518 CVD cases, balanced by equal number of non-CVD cases. The ROSE-generated samples may not capture true data distribution and models trained on this synthetic data may not calibrate well on real data. This affects clinical interpretability and not just technical performance, which limits the clinical utility of this work in general. I suggest the authors consider calibration plots to assess whether the models suffer from systematic probability miscalibration.

The SHAP plot for total cholesterol (please correct the spelling in the figure) in Figure 5 presents an interesting observation: that the relationship between total cholesterol and CVD prediction is non-linear and that there may be interaction effects. For example, age, medications, etc.). It is curious that high levels of a well-established CVD risk factor would have lower impact on CVD prediction. I am concerned that the model did not learn clinically meaningful relationships or that the synthetic data balancing introduced significant perturbations in the underlying relationship. This raises concerns about the clinical validity of other feature relationships identified by the models.

Major Concerns and Suggestions

Outcome Definition (Self-Reported CVD): The primary outcome is based entirely on self-reported diagnoses. This introduces the risk of recall bias and misclassification. Please provide further justification for this approach and discuss its limitations more explicitly in the Discussion section. If any objective biomarkers (e.g., ECG findings, clinical labs) could have been used as validation or proxy indicators, please consider this or explain their exclusion.

Cross-Sectional Design and Causal Inference: As the data are cross-sectional, causality cannot be inferred. Some language in the conclusions suggests predictive utility that may overreach. Please temper these statements to avoid overstating implications for clinical practice.

Model Calibration: While discrimination metrics (e.g., AUROC, accuracy) are reported, calibration metrics (e.g., Brier score, calibration plots) are absent. Given the potential application to risk prediction, model calibration is essential. Please provide this analysis or justify its omission.

Missing Data and Complete-Case Analysis: A complete-case analysis was used for dietary variables with over 30% missingness, which may bias the sample. Please include: A comparison of included vs. excluded participants. A sensitivity analysis using imputed dietary variables (if feasible).

Class Imbalance and ROSE Oversampling: ROSE was used to address class imbalance in the training set. While this is appropriate, please clarify: Whether the test set was untouched (i.e., no synthetic sampling). How the oversampling strategy might affect model generalizability or introduce overfitting.

Lack of External or Temporal Validation: All models were trained and tested on the same time span. Please consider: Performing a temporal validation (e.g., training on 2017–2020, testing on 2021–2023) to assess if indeed changes in post-pandemic dietary patterns exist as you claimed. Include this limitation in the discussion if such validation is not possible.

Feature Selection Justification: The rationale for selecting 30 features via Recursive Feature Elimination is unclear. Please provide a performance curve to show how predictive power varies with the number of features.

Model and Code Availability: Given that NHANES is a public dataset and the study emphasizes interpretable and reproducible ML, I strongly recommend the authors share their model code and analysis pipeline via a public repository such as GitHub. At a minimum, this should include preprocessing scripts (e.g., imputation, feature selection), Final model training code for all five algorithms, Hyperparameter settings, Code to generate LIME and SHAP plots.

This aligns with *PLOS ONE*'s open science policies and would enhance the transparency, reproducibility, and utility of the study to the wider research community.

Minor Comments and Suggestions

Figures and Interpretability Tools: The manuscript references SHAP and LIME figures but could benefit from more in-text interpretation of these visualizations, particularly regarding individual-level insights.

Typographical and Stylistic Errors: Several typographical errors are present (e.g., “wodel” instead of “model”) and should be corrected throughout the manuscript.

Public Health Relevance: Consider expanding on how your findings might guide nutritional policy or targeted interventions, particularly in vulnerable populations.

Conclusion:

This manuscript presents an important and novel contribution to the intersection of nutrition, machine learning, and cardiovascular risk prediction. However, methodological clarifications, additional analyses, and a more cautious interpretation of findings are needed before the manuscript can be considered for publication.
